# Evolutionary traces decode molecular mechanism behind fast pace of myosin XI

**DOI:** 10.1186/1472-6807-11-35

**Published:** 2011-09-26

**Authors:** Divya P Syamaladevi, R Sowdhamini

**Affiliations:** 1 National Centre for Biological Sciences, UAS-GKVK Campus, Bellary Road, Bangalore 560 065, India; 2 Sugarcane Breeding Institute(SBI-ICAR), Coimbatore, Tamil Nadu 641 007, India

## Abstract

**Background:**

Cytoplasmic class XI myosins are the fastest processive motors known. This class functions in high-velocity cytoplasmic streaming in various plant cells from algae to angiosperms. The velocities at which they process are ten times faster than its closest class V homologues.

**Results:**

To provide sequence determinants and structural rationale for the molecular mechanism of this fast pace myosin, we have compared the sequences from myosin class V and XI through Evolutionary Trace (ET) analysis. The current study identifies class-specific residues of myosin XI spread over the actin binding site, ATP binding site and light chain binding neck region. Sequences for ET analysis were accumulated from six plant genomes, using literature based text search and sequence searches, followed by triple validation *viz*. CDD search, string-based searches and phylogenetic clustering. We have identified nine myosin XI genes in sorghum and seven in grape by sequence searches. Both the plants possess one gene product each belonging to myosin type VIII as well. During this process, we have re-defined the gene boundaries for three sorghum myosin XI genes using fgenesh program.

**Conclusion:**

Molecular modelling and subsequent analysis of putative interactions involving these class-specific residues suggest a structural basis for the molecular mechanism behind high velocity of plant myosin XI. We propose a model of a more flexible switch I region that contributes to faster ADP release leading to high velocity movement of the algal myosin XI.

## Background

Myosin is an actin based motor protein that generates motion using chemical energy released through ATP hydrolysis. Myosins play many important roles within plant cells such as organelle trafficking [1,2], remodelling [3,4], and inheritance [5]. They are also known to be involved in development of various plant parts like root hairs, pollen *etc *[6,7]. Though there are around 24 classes of myosins reported in eukaryota [8], only three classes - class VIII, XI and XIII are seen in plants. The similarity of plant myosin sequences with animal and fungal class V myosins [9], derived from phylogenetic analysis, suggests a common ancestor from which plants and Opisthokonts [10] might have evolved.

Myosins, in general, function through an ATP hydrolysis cycle by converting the hydrolysis energy to allosteric conformational changes within the motor head as well as the neck region leading to motion. Swinging cross-bridge hypothesis, proposed by H. E. Huxley, has been the most popular model to explain the molecular mechanism of energy transduction in myosins. Numerous biochemical and biophysical experiments thereafter helped to improve it to the present day swinging lever arm model [11], according to which, immediately after the release of ADP from the previous cycle, ATP binds to myosin head that is in an actin-bound post-stroke conformation. Upon hydrolysis of ATP, myosin head gets transformed to pre-stroke conformation which is actually actin unbound form. Upon rebinding to the next actin molecule, Pi is released first and the ADP bound myosin head changes conformation from weak to strong actin binding state. This will be followed by ADP release and conformational changes at the head domain back to post-stroke state where myosin strongly interacts with actin.

In plants, from algae to angiosperms, the high velocity cytoplasmic streaming (of the range 40-60 μm/s) is driven by myosins [12]. Myosin XI, found in plants, is the fastest known motor and it moves processively along actin filaments towards the plus-end, performing cellular functions like cytoplasmic streaming and vesicle transport [1,2]. Myosin XI is architecturally similar to class V myosin in animals, with a motor head followed by six IQ motifs, a coiled-coil and a globular cargo-binding domain called DIL [10]. Myosin XI, just like myosin V, functions as a dimer formed through coiled-coil interaction between the α-helical tail regions of the monomers. Both the classes have comparable step size (an average of 35nm) and same directionality towards the plus end of actin filament.

Due to the difficulties in crystallization of actin-myosin complex, the actual actin binding residues are not known even in well-characterized myosins like myosin II and V. However, docking studies have revealed the actomyosin interface residues (see ref. [13] for a review). Actomyosin interface is extensive in the rigour state because of the interaction of a single head with regions on two adjacent actin molecules. Rigour state contact between actin and myosin head can be divided into four regions: a large primary binding site on the face of actin, which on three sides, is flanked by three additional sites from surface loops [14]. In this study, using the Evolutionary Trace method, we have identified crucial residues at the actin binding site, at the ATP binding domain and at the beginning of neck region that could contribute to the fast release of ADP and the high velocity. During the process of accumulation of myosin XI sequences from plants, we have recognised nine Myosin XI sequences from sorghum and seven from grape through genome-wide survey and gene prediction.

Algal myosin XI, isolated from *Chara corallina *[15], slides F-actin *in vitro *at a speed equivalent to cytoplasmic streaming speed of 40- 60 μm/s, which is 10 times the speed generated by myosin V [16,17]. Studies with tobacco myosin XI heavy isoform, by Tominaga and coworkers, revealed that single myosin XI molecules move at velocity 7 μm/s along the actin and generate relatively smaller force, in the order of 0.5 pN [8] much smaller than the force generated by muscle myosin II [18] and by myosin V [19-21]. In an attempt to elucidate the mechanism of this fast movement of *Chara *myosin, Ito and co-workers measured its kinetic properties [22]. The rate constant of ADP dissociation from actin-motor domain complex of *Chara *myosin XI was estimated to be more than 2800 s^-1 ^and the rate constant of ATP-induced dissociation of motor domain from the actin was 2200 s^-1 ^at a physiological concentration of ATP. The estimated time spent on actin, in strongly bound state, was estimated to be <0.82ms. This value is the shortest among known values for various myosins and it has a duty ratio of <0.3 and a V_max _of actin-activated ATPase activity of 390 s^-1 ^[22]. ADP release, which is the rate limiting step in all other myosin types, is dramatically accelerated in this plant myosin. Most of the myosins possess positively charged residues on loop 2 where as Chara myosin has no net positive charge on loop2. Instead, positively charged residues are harboured on loop 3. Ito and coworkers investigated the effect of positively charged residues on the loops at the actin binding region and provided evidence for its partial role on the high velocity movement through mutation studies [23]. Still, the sequence signatures that lead to the specialization of these myosins as the fastest motors and the actual molecular mechanism of such a rapid process are not known completely. In this study, based on sequence analysis and molecular modelling, we propose that the sequence signatures at the switch I region, the ATP-binding site and the neck region as partly responsible for the observed high rate of ADP release, which in turn lead to the specialization of these myosins as the fastest ones.

## Methods

### Sequence search and validation

**Figure 1:**
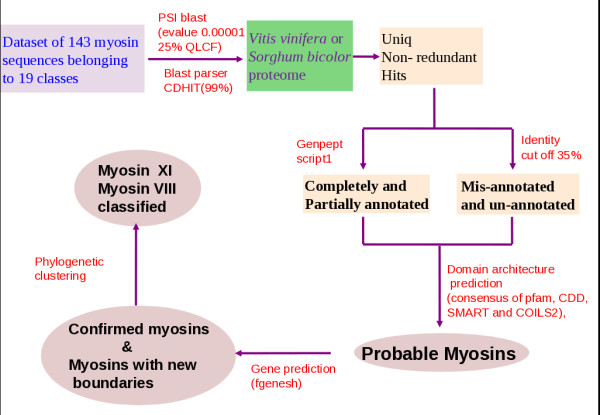
**Schema of extensive sequence search in two plant genomes: sorghum and grape**.

A database of 143 myosin sequences belonging to 19 different classes was developed through text search in NCBI and by consulting a database of myosins - The myosin home page (http://www.mrc-lmb.cam.ac.uk/myosin/myosin.html). The members of this database were used as queries in PSI-BLAST against sorghum and grape genomes. A relatively relaxed E-value cut-off of 10^-5 ^and a query length coverage filter of 25% were used in PSI-BLAST in order to retrieve even distantly related myosins. Hits were filtered for myosins through a three-fold validation procedure. In the first level, string-based scripts were used to gather completely or partly annotated myosin hits (Category 1) from respective GENEPEPT flat files. Mis-annotated or unannotated hits (Category 2) were gathered by using perl scripts based on the percentage identity of the hit with the query sequence. An identity cut-off of 35% was used here. The two categories of myosin hits obtained from sorghum and grape were checked for their domain architecture using PFAM [24], CDD [25], SMART [26] and COILS2 [27] servers. The consensus domain architecture, after consulting the domain repositories, was obtained for each of the validated hits. This forms the second level of filtering where false positive hits that deviate from the general domain architecture of myosins are eliminated. Such myosins were next subjected to gene prediction using fgenesh program [28]. Wherever the NCBI annotations were partial, full-length genes were obtained through fgenesh program and the gene boundaries are re-defined. The final sequence set obtained after these filters were annotated based on the co-clustering of motor domain (on the basis of sequence similarities) with representative sequences from 19 known myosin classes using neighbour-joining method of clustering in Phylip package [29]. This representative sequence dataset, obtained from 19 classes of myosins, were also seeded with representative sequences from three non-myosins - kinesin, ATPase and helicases - as outgroups to ensure the phylogenetic relationship of putative myosins with known myosins. This method of genome scan and validation is pictorially represented in a flowchart [Figure 1].

### Evolutionary Trace (ET) analysis

Class-specific residues were identified using ET method for myosin V and XI members. Multiple sequence alignment for 30 sequences from 10 genomes was done using CLUSTALW [30]. ET analysis was initially guided by Trace Suite II [31]. Sequences were divided into three parts based on domain architecture: 1) N-terminal SH3-like domain 2) motor domain and 3) light chain binding neck region. Class-specific residues for the motor domain and light chain binding neck domains were mapped on to myosin V crystal structure (PDB ID: 2DFS) and clustering of conserved residues were analysed.

### Molecular modelling of algal myosin XI

Homology model of the algal myosin XI (*Chara corallina*) was obtained using MODELLER [32]. Myosin V from chicken (PDB code: 1W7J) was used as a template [33]. Sequence identity between algal and chicken myosin head domains was 41%. Further, the predicted secondary structures of algal myosin head were comparable with the secondary structures identified in the template (chicken myosin head), except for fine-tuned boundaries. The best energy model from a set of 20 models, generated by MODELLER, was passed through various validation processes *viz*. WHATIF [34] and VERIFY-3D [35]. The model was energy minimized to relieve any inherent strain in the model and to obtain the most favourable conformation including side chains. The energy minimized structures were used to visualize the class-specific residues using PYMOL [36].

## Results and discussion

### Sequence search and validation identify putative myosins in soghum and grape

**Figure 2:**
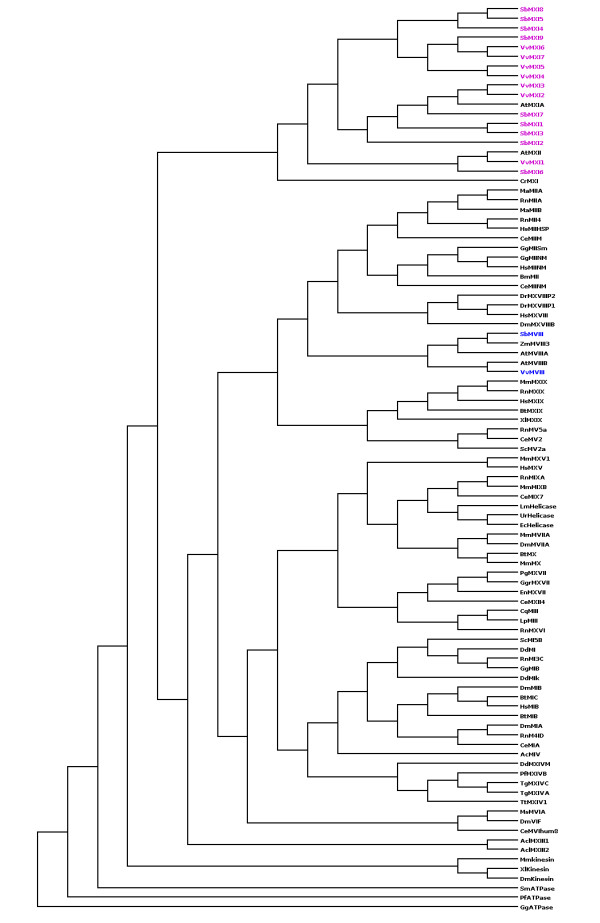
**Unrooted phylogenetic tree showing the evolutionary relationship of putative myosins from sorghum and grape**. Myosin XI types are in pink colour and Myosin VIII types are in blue.

Proteomes of a monocot and a dicot (*Sorghum bicolor *and *Vitis vinifera *) were scanned for myosin types using PSI-BLAST preceding a three-fold validation as mentioned in Methods. Additional myosin class V and XI sequences were obtained from three different plant genomes: *Arabidopsis thaliana*, *Oryza sativa and Zea mays*. Motor domains of validated hits, including those with new predicted boundaries (Table 1) were subjected to clustering (Figure 2) so as to annotate their subfamily types and classified them as belonging to myosin VIII or XI subfamily. Sorghum genome harbours 10 myosin genes distributed in seven chromosomes, whereas grape genome contains only eight myosins out of which seven are myosin XI isoforms (Figure 2) spread over eight chromosomes (Table 1). Sorghum has one type VIII and nine type XI myosin genes. For three of the predicted myosin XI in sorghum, we have re-defined the gene boundaries using fgenesh gene prediction program. We assigned new boundaries to XP_002458397 in Chromosome 3 and merged two partial genes (XP_002440148 & XP_002440149) into a single gene with complete domain architecture of type XI myosin.

**Table 1:** Details of confirmed myosins from sorghum and grape after multi-fold validation

Organism	Id/Acc number	Chromosome region	Gene ID	Protein length	%ID*	Annotation	Motor domain
VvMXI1	XP_002281615	Chr4 1597682 - 1630481	100254166	1517	55	XI	54-730

VvMXI2	XP_002285579	Chr5 10420215 - 10531891	100259730	1513	61	XI	57-734

VvMXI3	XP_002268099	Chr7 8436820 - 8456586.	100256590	1518	62	XI	63-739

VvMXI4	XP_002274978	Chr9 1674607 - 1696216.	100266890	1535	77	XI	66-743

VvMXI5	XP_002279028	Chr11 1332352 - 1348606	100243893	1586	75	XI	113-790

VvMXI6	XP_002263591	Chr14 360176 - 377881	100243373	1587	70	XI	113-790

VvMXI7	XP_002263354	Chr7 6130176 - 6182090	100265237	1204	59	XI(DILminus)	126-803

VvMVIII	XP_002281748	Chr19 3770898 - 3797194	100241905	1229	30	VIII	210-888

SbMXI1	XP_002463461	Chr1 186074 - 205207	8061371	1557	56	XI	85-810

SbMXI2	XP_002466464	Chr1 7057755 - 7066246	8059553	1464	60	XI	57-733

SbMXI3	XP_002461898	Chr2 14576408 - 14597301	8057935	1497	59	XI	56-732

SbMXI4	XP_002458046	Chr3 52499134 - 52510404	8078219	1529	70	XI	108-783

SbMXI5	XP_002458397	61226792 - 61232762 new:61219493-61232763	8069786	499 1498	49	XI	56-952

SbMXI6	XP_002452906	Chr4 64687149 - 64704955	8082670	1520	53	XI	51-731

SbMXI7	XP_002453038	Chr4 66880218 - 66891041	8056444	1347	60	XI	1 to 566

SbMXI8	XP_002440148 & XP_002440149	Chr9 56017722 - 56023175 & 56024467-56037464 New:56017040-56037361	8061068 & 8061069	610 & 1399 2176	51	XI	113-796

SbMXI9	XP_002436758	Chr10 8229550 - 8241900.	8057883	1539	66	XI	67-743

SbMVIII	XP_002467046	Chr1 19620879 - 19630164	8067384	1196	32	VIII	190-868

* Full-length percentage identity with *Arabidopsis *myosin XI

Both sorghum and grape genomes contain only one copy of myosin VIII type in chromosome 1 and 19, respectively (Table 1).

**Figure 3:**
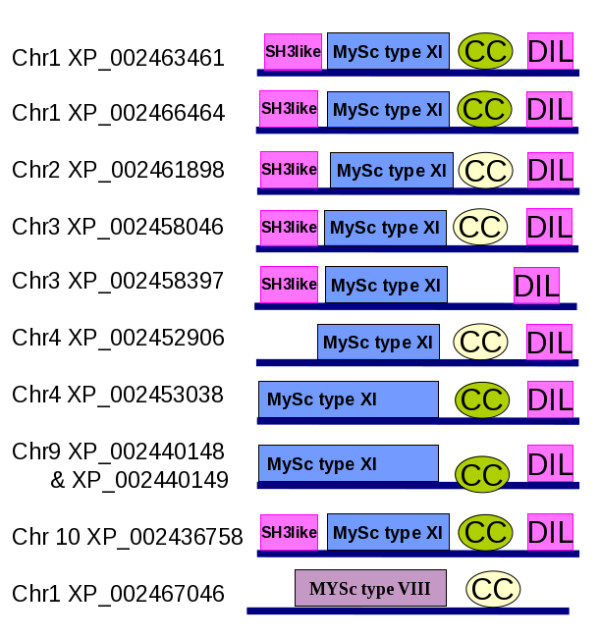
**Domain architectures of putative myosins from Sorghum**. XP_002458397, on Chromosome3, was a hypothetical partial gene and assigned a new gene boundary according to fgenesh. The domain architecture corresponds to the new gene boundary. XP_002440148 & XP_002440149 of chromosome 9 are two adjacent incomplete genes, which are merged to a single gene with new gene boundaries and the corresponding domain architecture is shown here. 'CC' denotes the coiled coil domain. Green colour indicates that it is detected by domain prediction servers and off-white colour indicates that it was predicted using COILS program. 'Chr' indicates the chromosome on which the gene is located.

**Figure 4:**
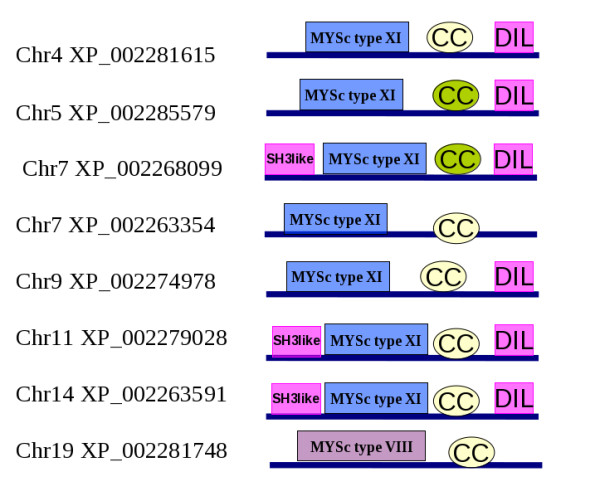
**Domain architectures of putative myosins from grape, their chromosome and the domain architecture**.

The domain architecture predicted for these new myosins from sorghum and grape are shown in Figures 3 and Figure 4, respectively. All the myosin XI gene products from sorghum and grape followed the common domain architecture of motor head-coiled coil-DIL except one, namely VvMXI7 (Table 1). Myosin VIII from both the genomes had the two-domain architecture of motor head followed by coiled coil. Myosin VIII sequences do not possess features of a compact domain at the N-terminal region before the head domain, where as many of the myosin XI types have an N-terminal SH3-like domain that is involved in interaction with actin [37]. The effects of presence or absence of the co-existing domains on their motility and cellular behaviour are yet to be understood.

### Class-specific residues in myosin XI lines actin binding site

**Figure 5:**
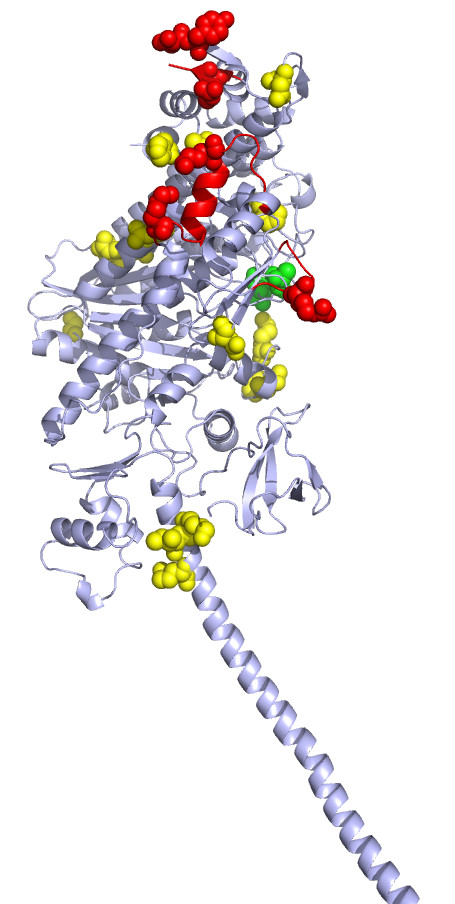
**Class-specific residues (shown as spheres) mapped on to the structure of myosin V (PDB Id: **2DFS). Red spheres are the class-specific residues at the putative actin binding site. Few class-specific residues observed near the actin binding site and neck region are shown in yellow. The green spheres are the class-specific residues at the ATP binding site.

The full-length of myosin XI shares 31% overall identity with myosin V and the motor head domains alone share a high sequence identity (above 40%). There is a high level of sequence conservation between the two classes at the active site residues. Nevertheless, the two classes differ considerably at functionally important sites and mechanistic details, according to the literature (as mentioned in Introduction). Myosin XI head contains 21 class-specific residues, out of which six appear on the actin binding sites. Known actin binding sites on myosin II fall into three regions: A primary binding site comprising of a helix-loop-helix and an adjacent helix, a secondary site which is a charged loop and a tertiary site lying at the front or nose of the myosin molecule [13]. The corresponding regions on myosin V are marked on the 2DFS structure in red color (Figure 5). All the three sites contain class-specific residues. This implicates the possible involvement of these residues in imparting the differential actin-binding strength of the two types of myosins.

It was recently shown that the ATP-induced motor head dissociation in myosin XI occurs at a very fast rate of 2200s-1, resulting in stunningly fast movement of myosin XI [22]. In our analysis, we identified two myosin XI-specific residues in the primary binding site (helix-loop-helix) that corresponds to K502 and K514 of chicken myosin V. In myosin XI, K502 and K514 are mutated in a class-specific manner to proline and methionine, respectively. This may introduce a structural kink and local rigidity to the loop and disrupt the charged interaction of the primary binding site to actin. This can help the myosin XI motor head to get unbound sooner from the actin upon ATP binding after the release of Pi and ADP in the previous ATP-hydrolysis cycle.

Apart from the three key actin binding sites, surrounding residues may also get involved in actin binding. Few of the class-specific residues observed near the actin binding site may also contribute to binding strength (yellow spheres at the actin binding -region in Figure 5). The second actin binding site on myosin XI also harbours a class-specific residue: Lysine, but the mutation R542K seems to be too subtle to introduce a difference in the interaction with actin.

### Putative involvement of two myosin XI specific residues in switch I flexibility

**Figure 6:**
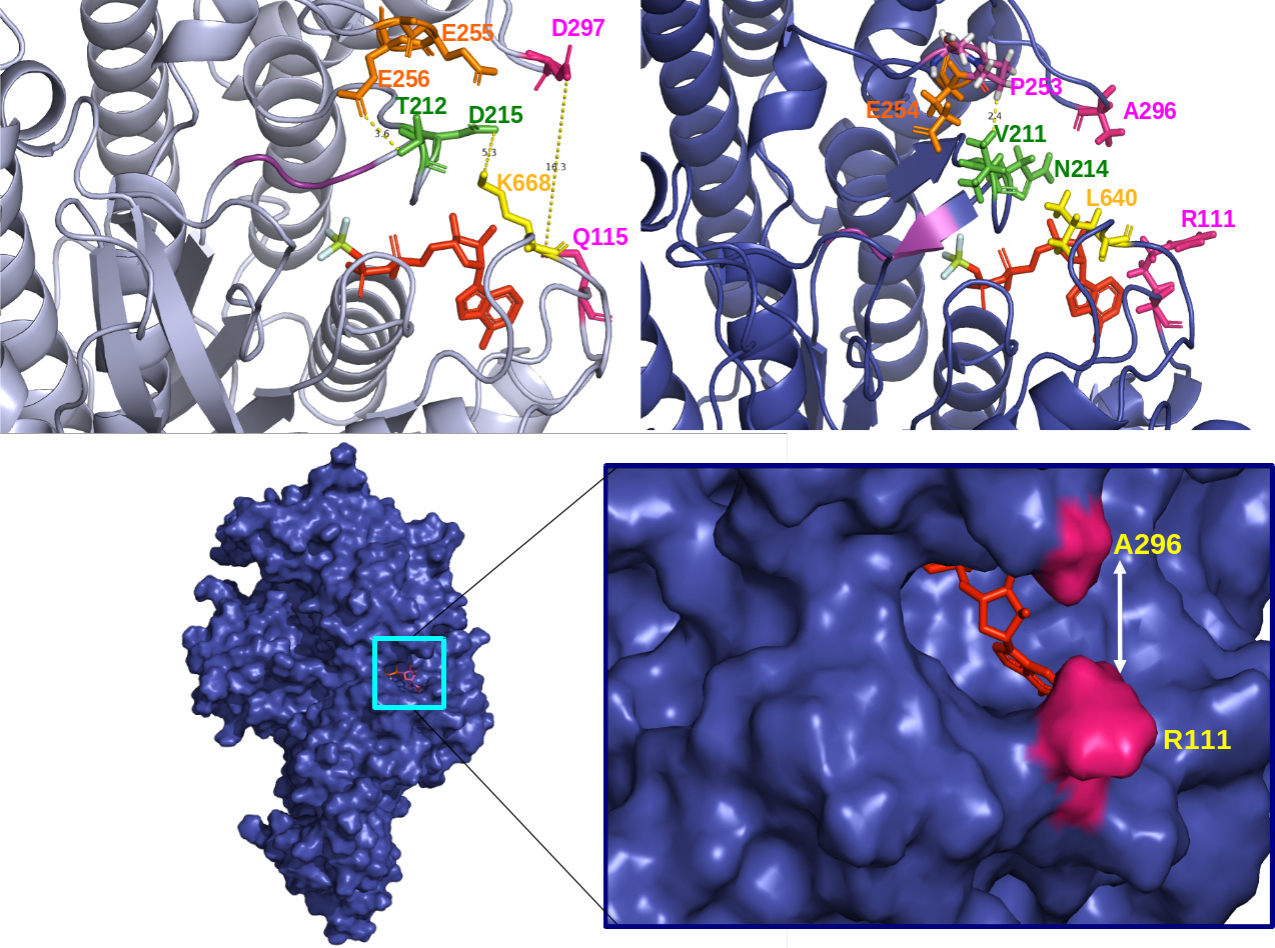
**Residues lining the ATP binding site of A) chicken myosin V and B) *Chara *myosin XI model are shown in stick representation C) Structural model of *Chara *myosin XI showing the ATP binding site and the shutter residues coloured in pink and D) a close-up of shutter region**.

The switch I region, which drives an open-close conformational change in the myosin head during ATP hydrolysis [38], has two class-specific residues (shown in green in Figure 5). One of these is Asn 214 (algal myosin XI numbering), corresponding to Asp215 of myosin V. In myosin V, Asp215 has a favourable charged interaction with Lys668, whereas in myosin XI, due to changes of Asp215 to Asn214 and Lys688 to Leu640, this stabilizing interaction is likely abolished which could lead to a more flexible switch I region. These class-specific residues are mapped on the three-dimensional model of algal myosin head (Figure 6A). Since the movement at the switch I region is intimately associated with γ-phosphate binding, coordination of Mg^2+ ^and there by ADP binding and release, we propose a more flexible switch I region in myosin XI that might facilitate an early or easy release of ADP.

In Myosin V, Thr 212 is located close to a Glutamate triad (Glu254, Glu255 and Glu256) and it stabilizes the switch I through a favourable interaction with Glu256. In plant myosin XI, there is an interesting co-evolution at this location, where a spatially proximate Thr212 is replaced by Val and Glu255 by Pro (Figure 6A &6B). As a result of this, stabilizing polar interaction of Thr212 with Glu255 observed in myosin type V appears obsolete in myosin XI. A new possible interaction between Valine and Proline seems to be stabilizing the switch I region of myosin XI (Figure 6A, 6B).

### Shutter residues might make the ATP binding cavity closure inefficient in *Chara coralina*

A closer investigation of the ATP binding site (Figure 6C &6D) of the myosin V crystal structure, reveal two mutations in plant myosins that would probably decide the opening and closing of the small cleft at the ATP binding site. Asp297 is located in an upper loop at the face of the cleft and has a potential polar interaction partner Gln115 located in a lower loop of myosin V. In Figure 6A, this interaction is highlighted through a dotted line in the near-rigour-open conformation, as seen in the crystal structure of myosin V. This interaction can form a vertical shutter for the ATP binding cavity. These 'shutter' residues are Ala296 and Arg111 in myosin XI from *Chara coralina*, which are stabilised by weak dispersion forces, and hence the closure of the cleft may become inefficient in *Chara *myosin.

### Weakened interaction between the neck and the converter domain affects lever arm tilting

We have also examined the neck region (also termed as 'lever arm') for class-specific residues and the conservation of amino acid types. We identified a cluster of three class-specific residues, two of which (Ile762 and Lys766, chicken myosin V numbering) have potential interactions with the converter domain in the case of myosin V (coloured yellow in Figure 5, Additional files 1 and 2).

(a) Ile762 (in chicken myosin V, please see Additional file 2) at the beginning of the neck region, is embedded between two hydrophobic residues - a highly conserved Leu714 and a conserved hydrophobic residue at position 735 - of the converter domain forming a "hydrophobic triad". The corresponding residue of Ile762 in plant myosin XI neck region is a charged arginine that possibly weaken the hydrophobic interaction of the N-terminal neck with the converter domain.

(b) Spatially close to this "hydrophobic triad" in myosin V (please see Additional files), the side chain of Lys766, the second class specific residue, interacts with the backbone oxygen of Val713 located at the converter domain by forming a hydrogen bond. In plant myosin XI, Lys766 is replaced by a Valine in a class specific manner. As a result of this change, a weak hydrophobic interaction alone could prevail between the neck and the converter domains of myosin XI (Additional file 1). Such amino acid differences could provide higher conformational flexibility to plant myosins in tilting the lever arm (neck region) with ease, which in turn is associated with a faster ADP release and actin dissociation. The third class-specific residue (not shown in Additional file 1) is likely to interact with SH3 domain.

## Conclusion

**Figure 7:**
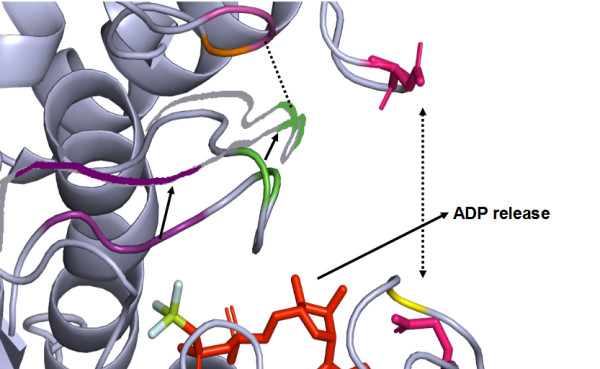
**A model depicting the possible mechanism of ADP exit in plant myosin XI and the associated conformational changes**.

For a processive myosin to move at high speed on the actin filament, the best way is to strike an optimal duty ratio so that the dwell time is just sufficient to locate the next binding site on actin. ADP release is the rate limiting step of ATP hydrolysis cycle and the associated movement of myosin along the actin filament. One of the possible ways to attain speed is to hasten the ADP release process. We propose that altered sequence conservation patterns could redefine structural interactions and impart differences in speed. A poorly closed ATP binding cavity, together with a more flexible switch I region, is compelling to think of a model for faster release of ADP in plant myosins. From our sequence analysis, the switch I region bears unique sequence signatures in the fast-acting myosin. As shown in Figure 7, this region may be pulled away from the ADP binding site resulting in the loss of co-ordination of Mg^2+^, which in turn would make the ADP release easier. As the gating loops of the ATP binding cavity in Myosin XI are not directly interacting, due to class-specific amino acid differences, the closing of the cavity may be for a very short period and this may facilitate the release of ADP smoothly without any further delay.

From this comparative sequence analysis, we could recognise most crucial residue changes that may be responsible for decreased/altered affinity of myosin XI to the actin and rapid ADP release in comparison with myosin V. A molecular mechanism of fast movement of myosin XI could be proposed: As long as ADP is bound to the myosin head, actin binding is stabilized through numerous weak interactions. Due to the enhanced flexibility of the switch I region, ADP gets released very fast after hydrolysis. Release of ADP permits the next ATP to bind and subsequently dissociate the acto-myosin complex through suitable conformational changes at the actin-myosin interface, which would further be enhanced due to the absence of few stabilizing charged interactions. Meanwhile, since the neck region in myosin XI interacts only poorly with the converter domain, the neck flexibility is also high which might assist in faster catalysis and binding events at the head domain.

## Authors' contributions

DPS carried out the study and performed the computational analysis. RS participated in the analysis and comparative sequence analysis. DPS drafted the manuscript and RS has improved to provide the final manuscript. All the authors have read and approved the final manuscript.

## Supplementary Material

Additional file 1 **The class-specific residues of myosin XI are mapped over the neck region of myosin V crystal structure**. Click here for file

Additional file 2 **Class-specific residues (I762K and K766V) at the neck region are shown on the multiple sequence alignment**. Click here for file
