# Supplementary figures and images for: Evolutionary traces decode molecular mechanism behind fast pace of myosin XI
Source: BMC Struct Biol. 2011 Sep 26;11:35. doi: 10.1186/1472-6807-11-35 (PMC3209465; doi:10.1186/1472-6807-11-35)

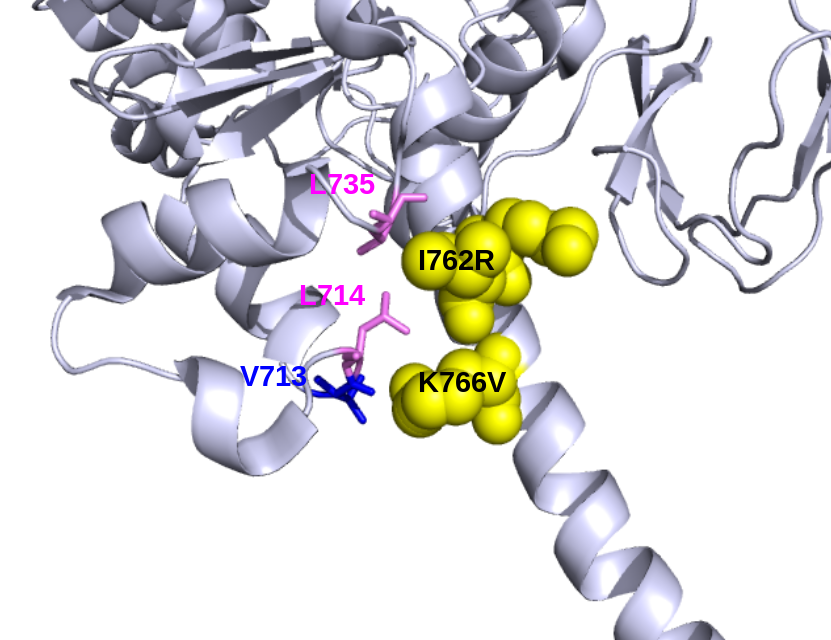

Supplement: Additional file 1 — The class-specific residues of myosin XI are mapped over the neck region of myosin V crystal structure. [file 1472-6807-11-35-S1.PNG]

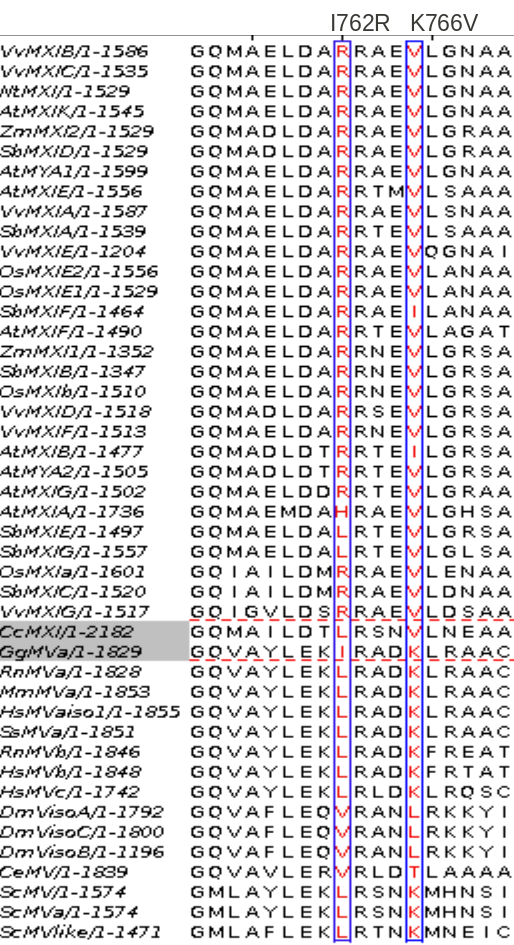

Supplement: Additional file 2 — Class-specific residues (I762K and K766V) at the neck region are shown on the multiple sequence alignment. [file 1472-6807-11-35-S2.PNG]
